# Supplementary material for: Genetic comparisons of interleukin-17 reveal a framework for complex signaling evolution
Source: bioRxiv. 2026 Apr 14:2026.04.13.718218. Preprint. [Version 1] doi: 10.64898/2026.04.13.718218 (PMC13105070; doi:10.64898/2026.04.13.718218)
Supplement: Supplement 3 [file media-3.zip › Supplemental files for paper/ERC/IL17xIL17_comparisons/IL17A_IL17RB.pdf]

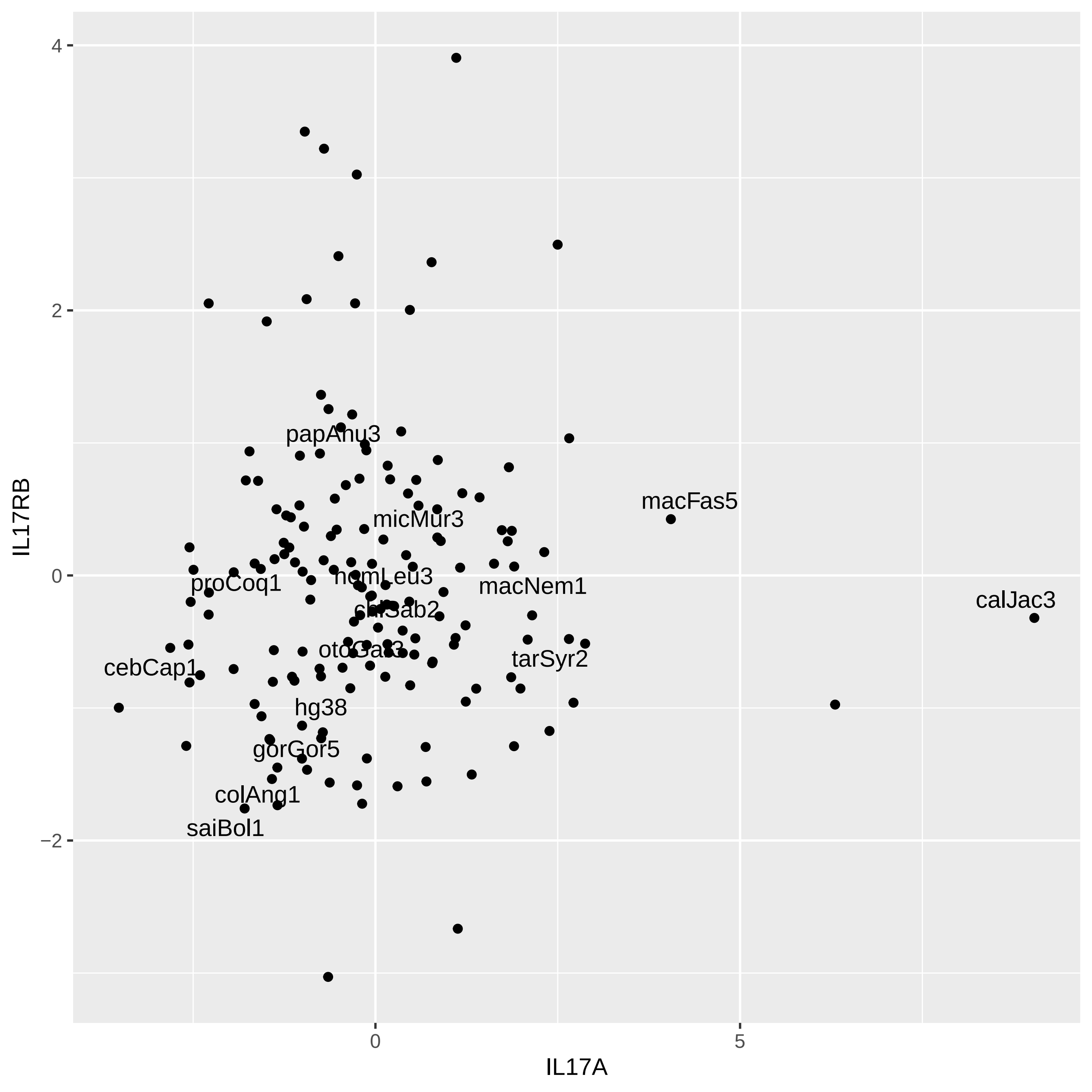
IL17RB
IL17A
4
2
0
−2
0
5
papAnu3
macFas5
micMur3
proCoq1
nomLeu3
macNem1
calJac3
chlSab2
otoGar3
tarSyr2
cebCap1
hg38
gorGor5
colAng1
saiBol1
